# Supplementary material for: Co-developed implementation guidelines to maximize acceptability, feasibility, and usability of mobile phone supervision in Kenya
Source: Glob Ment Health (Camb). 2023 May 23;10:e31. doi: 10.1017/gmh.2023.23 (PMC10579659; doi:10.1017/gmh.2023.23)
Supplement: Supplementary file 1 [file S2054425123000237sup.zip › S2054425123000237sup001.docx]

**BASIC Pamoja Tunaweza Mobile Phone Supervision**

**Best Practices**

***Mobile Phone Supervision Meeting: Soon after counselors have finished the Pamoja Tunaweza (PT) training. This will happen at the same time as Coaching Meeting 2.*** Now that counselors have been trained, they probably have additional questions for you that are important for finding solutions to support mobile phone supervision for PT.

Date of Meeting: ___________________________________ Site: _________________________________________

Counselor IDs: ____________________________________________________________________________________

**Planning Solutions to Support Mobile Phone Supervision**

There are 4 goals for this meeting. Goals are listed below, with the strategies/solutions used by counselors in Steps 1-5; however, each new community can always identify their *own* new solutions. Ideally each solution plan that will support mobile phone supervision should be tailored to fit the unique community. In your conversation, please consider the unique aspects (urban or rural setting etc.) that may influence the solutions that will be best for them. For example, more urban schools may have fewer problems with network connection.

***Goal A*: Explain to counselors that we will use in-person and mobile phone supervision together to ensure they are supported through PT delivery.**

1. We will provide **in-person supervision at least four times during PT delivery**. This will be at the beginning, middle, and end of PT groups.

- Supervision will be provided through mobile phone throughout the PT program. Counselors should feel free to use their mobile phones to communicate with their supervisors for support at any point during the program.

1. If you need additional support, you may **request that your supervisor comes** for additional in-person supervision visits.

- Other counselors have requested support when facing challenges with certain topics or clarifying the goals of the PT program.

***Goal B*: Ensure counselors get all the information and support that they need through mobile phone supervision.**

1. Supervisors and counselors should save each other’s **contact information**.

- Counselors should also save each other’s contact information so that they can communicate and plan for PT groups together.
- If counselors change phone numbers, then they should communicate that to their supervisors and counselors, who will update their contact information.

1. Supervisors may use **phone calls, SMS, and WhatsApp messages** to communicate with counselors.
2. Supervisors will **communicate important messages to the entire group** via group calls, group SMS, or WhatsApp groups.

- Supervisors will message counselors individually for personal matters, and counselors can feel free to reach out individually for personal support.

1. Supervisors will communicate in **suitable language**.

- Supervisors will use the language of the counselors choosing (e.g., Swahili or English).

1. Counselors shall be supplied with **airtime** and can request that supervisors call them to preserve their own airtime.

- Counselors can “reverse call,” “flash,” or send a “please call me message” to their supervisors when their airtime is low.

1. Counselors should **ensure their phones are charged** for supervision.

- Other counselors have used power banks, solar power, or back-up batteries to ensure their phones are charged for supervision.

***Goal C*: Plan for any challenges with network connection.**

1. Counselors should ensure their **phones are updated** for the best network connection.

- Counselors can update their phones by switching off and back on.
- Counselors should ensure their SIM cards are updated (example: 3G or 4G) for strongest network connection.

1. Counselors should **identify places near their schools with strong network connection**.

- If counselors have multiple SIM cards, they should check for the strongest connection on either network.

***Goal D*: Decrease distractions and disruptions during mobile phone supervision.**

1. Counselors should find a **private and quiet place for supervision** phone calls.
2. **Supervisors will notify counselors in advance** when they will be having mobile phone supervision.

- Counselors should notify supervisors if they need to reschedule supervision via message or call.

1. Counselors should **notify others that they will be taking a supervision** phone calls.

- Counselors shall delegate work responsibilities if necessary.
- Counselors should turn on “call waiting” so that they do not get other calls during supervision.

1. In the event of **weather and poor network, counselors shall SMS supervisors** to reschedule.

**Notes:**
