# Supplementary material for: Co-developed implementation guidelines to maximize acceptability, feasibility, and usability of mobile phone supervision in Kenya
Source: Glob Ment Health (Camb). 2023 May 23;10:e31. doi: 10.1017/gmh.2023.23 (PMC10579659; doi:10.1017/gmh.2023.23)
Supplement: Supplementary file 1 [file S2054425123000237sup.zip › S2054425123000237sup002.docx]

INSTRUCTIONS FOR PARTICIPANT:

**BASIC Semi-Structured Mobile Phone Supervision Qualitative Interview – Teacher Version**

*Thank you for being willing to do an interview with us. We will be asking you some questions about your experience using mobile phones to communicate with your supervisor in your role as a Pamoja Tunaweza counselor. This interview will take about* ***one hour*** *to complete and will be* ***audio recorded for the purposes of transcription only and also not to miss out on any important information you give us****. Your answers will help us learn how to better support Pamoja Tunaweza delivery in communities. There are no right or wrong answers, we want to hear your honest opinions. Your individual responses are confidential and will not be shared with your Head Teacher, anyone from your community, your teacher coach, or the Ace Africa supervisors. Your answers will not impact any funding or resources you receive from your community, school, or Ace Africa. Your responses will not be attached to your name or your community’s name. They will only be attached to a study ID number, and will only be viewed by the research team. Your responses will only be used to improve support for Pamoja Tunaweza. As a participant, you will receive 500 ksh for completing this interview.*

*For this interview,* ***we are interested in learning about your experience in the meeting you and your co-counselors had with your supervisor to discuss mobile phone supervision and ways to improve it.*** *During this meeting, your supervisor reviewed a mobile phone supervision worksheet and discussed potential strategies to improve mobile phone supervision with you and your co-counselors. I have brought one of these sheets for your reference. We will also be asking about how this meeting impacted your experience receiving mobile phone supervision. This may include phone calls, SMS, WhatsApp messages, or any other way of communicating with your supervisor with your phone about Pamoja Tunaweza supervision. Please think about* ***all*** *these activities when you’re answering questions about mobile phone supervision. We will be asking about what you liked about communicating with your PT supervisor through your mobile phone and the challenges you faced.*

***Interviewer note:*** *Throughout the interview, please remind the participant not to mention any specific names but to specify roles (e.g., Head Teacher, Deputy Teacher, Senior Teacher, Coach, Supervisor, ACE staff).*


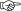


**PART 1**

**1. On a scale of 1-10, with 1 being not at all useful and 10 being extremely useful, please rate how useful having a meeting to discuss how to use the mobile phone for supervision and ways to improve it was in helping you communicate with your Pamoja Tunaweza supervisor over your mobile phone.**

| 1 | 2 | 3 | 4 | 5 | 6 | 7 | 8 | 9 | 10 | DK |
| --- | --- | --- | --- | --- | --- | --- | --- | --- | --- | --- |
| Not at all useful |  |  |  | Moderately useful |  |  |  |  | Extremely useful |  |

***Follow-up question:* You’ve just given me a rating of [the rating]. Tell me more about why you chose that?**

**2. How did this meeting impact how possible or feasible it was to communicate with your Pamoja Tunaweza supervisor over your mobile phone?**

**3. What strategies that you discussed with your supervisor were most helpful for you to receive mobile phone supervision in your community?**

**4. What strategies that you discussed with your supervisor were not helpful for you to receive mobile phone supervision in your community?**

**5. How did the airtime, that you discussed that you would be receiving during the meeting, help you communicate with your Pamoja Tunaweza supervisor about Pamoja Tunaweza supervision?**

***Follow-up question:* Was the amount provided enough for you to communicate with your Pamoja Tunaweza supervisor?**

**6. Are there any other supports that you needed for mobile supervision, but we were not able to provide?**

**7. Is there anything else about the meeting to discuss how to use the mobile phone for supervision that I haven’t asked about that you’d like to share?**

**PART 2**

*INSTRUCTIONS: Next, I want to ask some other questions about* *your experience using mobile phones to communicate with your supervisor in your role as a Pamoja Tunaweza counselor. When we talk about communicating with your supervisor using mobile phones, we mean any way you have communicated with supervisor in relation to Pamoja Tunaweza supervision. This may include phone calls, SMS, WhatsApp messages, or any other way of communicating with your supervisor with your phone about Pamoja Tunaweza supervision. Please think about* ***all*** *these activities when you’re answering questions about mobile phone supervision.*

**8. What do you like most about communicating with your Pamoja Tunaweza supervisor through your mobile phone about Pamoja Tunaweza supervision?**

**9. What is challenging or frustrating about communicating with your Pamoja Tunaweza supervisor over your mobile phone?**

***Follow-up question:* How did you overcome those challenges?**

**10. Is there anything else about receiving supervision by your mobile phone that I haven’t asked about that you’d like to share?**
