# Supplementary material for: Co-developed implementation guidelines to maximize acceptability, feasibility, and usability of mobile phone supervision in Kenya
Source: Glob Ment Health (Camb). 2023 May 23;10:e31. doi: 10.1017/gmh.2023.23 (PMC10579659; doi:10.1017/gmh.2023.23)
Supplement: Supplementary file 1 [file S2054425123000237sup.zip › S2054425123000237sup003.docx]

*Pearson Correlations Between Acceptability, Feasibility, and Usability*

| Measure | Acceptability | Feasibility | Usability |
| --- | --- | --- | --- |
| Acceptability | 1 | 0.65 | 0.57 |
| Feasibility | 0.65 | 1 | 0.76 |
| Usability | 0.57 | 0.76 | 1 |
